# Food Grade Pimenta Leaf Essential Oil Reduces the Attachment of *Salmonella enterica* Heidelberg (2011 Ground Turkey Outbreak Isolate) on to Turkey Skin

**DOI:** 10.3389/fmicb.2017.02328

**Published:** 2017-11-28

**Authors:** Divek V. T. Nair, Anup Kollanoor Johny

**Affiliations:** Department of Animal Science, University of Minnesota, Saint Paul, MN, United States

**Keywords:** pimenta, essential oil, *Salmonella* Heidelberg, turkey skin, microscopy

## Abstract

*Salmonella* attached to the poultry skin is a major source of carcass contamination during processing. Once attached to the poultry skin, it is difficult to detach and inactivate *Salmonella* by commonly used antimicrobial agents since the pathogen is entrapped deeply in the feather follicles and the crevices on the skin. Essential oils could be natural, safe, and effective alternatives to synthetic antimicrobial agents during commercial and organic processing setup. The present study evaluated the efficacy of pimenta (*Pimenta officinalis* Lindl.) leaf essential oil (PEO), and its nanoemulsion in reducing *Salmonella* Heidelberg attachment on to turkey (*Meleagris gallopavo*) skin during simulated scalding (65°C) and chilling (4°C) steps in poultry processing. A multidrug resistant *S.* Heidelberg isolate from the 2011 ground turkey outbreak in the United States was used in the study. Results showed that PEO and the nanoemulsion resulted in significant reduction of *S.* Heidelberg attachment on turkey skin. Turkey skin samples treated with 1.0% PEO for 5 min resulted in >2 log_10_ CFU/sq. inch reduction of *S.* Heidelberg at 65 and 4°C, respectively (*n* = 6; *P* < 0.05). Similarly, skin samples treated with 1.0% pimenta nanoemulsion (PNE) for 5 min resulted in 1.5- and 1.8- log_10_ CFU/sq. inch reduction of *S.* Heidelberg at 65 and 4°C, respectively (*n* = 6; *P* < 0.05). In addition, PEO and PNE were effective in reducing *S.* Heidelberg on skin during short-term storage at 4 and 10°C (temperature abuse) (*n* = 6; *P* < 0.05). No *Salmonella* was detected in the dipping solution containing 0.5 or 1.0% PEO or PNE, whereas a substantial population of the pathogen survived in the control dipping solution. The results were validated using scanning electron -, and confocal - microscopy techniques. PEO or PNE could be utilized as an effective antimicrobial agent to reduce *S*. Heidelberg attachment to turkey skin during poultry processing.

## Introduction

Historically, *Salmonella enterica* serovar Heidelberg (*S*. Heidelberg) has been one of the common *Salmonella* associated with poultry and is frequently isolated from turkeys (Foley et al., 2008; Jackson et al., 2013). Being one of the most invasive of *Salmonella* serotypes in humans, the pathogen has surfaced to importance causing significant economic loss to the poultry industry and public health. In 2011, the consumption of contaminated ground turkey meat resulted in 136 human infections in 34 United States (CDC, 2011). In 2013, multidrug resistant (MDR) *S*. Heidelberg infections were linked to contaminated poultry products from a commercial processor in California (CDC, 2014a). Another outbreak caused by *S*. Heidelberg, including its MDR clones, was reported from a Tennessee correctional facility linked to consumption of contaminated poultry meat (CDC, 2014b). More recently, *S*. Heidelberg was ranked third among the most common etiological agents of human salmonellosis in the United States (CDC, 2013) and is currently an urgent threat to the United States food supply due to their high antibiotic resistance (AR) potential (Folster et al., 2012a,b; Shah et al., 2017)

Turkeys can harbor *S.* Heidelberg in their cecum without showing clinical disease (Poppe et al., 2005; Nair et al., 2016). The excretion of the pathogen through droppings can contaminate farm, and faulty evisceration step during processing could result in the contamination of turkey carcasses with *S.* Heidelberg (WHO, 2009). *Salmonella* attached to the carcass skin is a major source of poultry meat contamination during processing (Kim et al., 1996; Carrasco et al., 2012). During the scalding step, the turkey carcasses are immersed in water at 59 to 63°C for 50 to 125 s (FSIS, 2010) to up to a reported 65°C (Logue et al., 2003) to loosen the hair follicles (Carrasco et al., 2012). This step is considered as the first and critical step where a high likelihood of cross-contamination with pathogenic bacteria, including *Salmonella*, could occur (Russell, 2008; Carrasco et al., 2012). During chilling, the carcasses are immersed in cold water (4.4°C) where the chances of cross-contamination of carcasses with *Salmonella* is high (Nagel et al., 2013). The water uptake and swelling of the poultry skin during immersion chilling also expose deep channels and crevices on the skin, making the conditions favorable for bacterial attachment (Kim et al., 1996). The oily nature of poultry skin, penetration of *Salmonella* into the feather follicles, entrapment of *Salmonella* in the skin crevices, and the presence of high organic load on skin surface during scalding and chilling may reduce the effectiveness of chlorine and other commercially used synthetic disinfectants (Lillard, 1990; Kim et al., 1996; Yang et al., 2001; Nchez et al., 2002; Nagel et al., 2013). Along with these factors, various genetic mechanisms enable *Salmonella* to attach tightly to the skin of poultry carcasses (Salehi et al., 2017).

Recently, there is a tremendous interest in using natural antimicrobials as an alternative to synthetic chemicals against pathogens during food production (Burt, 2004; Lanciotti et al., 2004; Amalaradjou et al., 2010; Kollanoor Johny et al., 2010; Nair et al., 2015; Surendran Nair et al., 2016; Surendran-Nair et al., 2016). Among the emerging and widely researched alternatives, essential oils (EO) or their components are reported effective against foodborne pathogens such as *Salmonella, Campylobacter*, and *Escherichia coli* O157 *in vitro* and *in vivo* (Kollanoor Johny et al., 2008; Amalaradjou et al., 2010; Kollanoor-Johny et al., 2012a,b; Nair et al., 2014, 2015). The United States Food and Drug Administration (USFDA) has approved the use of selected EOs in food matrices (FDA, 2016) and therefore, their efficacy could be evaluated as safe and natural alternatives in commercial and organic poultry production systems (GPO, 2017). The use of EOs is also advantageous since they possess multiple active chemical sites to counteract pathogens using different mechanisms reducing the potential for the development of antimicrobial resistance against EOs (Venkitanarayanan et al., 2013; Surendran Nair et al., 2017).

Pimenta essential oil (PEO), commonly known as allspice oil, is extracted from the leaves of *Pimenta officinalis Lindl*. and is approved as Generally Recognized As Safe (GRAS; CFR-Title 21: Part 182, Sec. 182.20) compound by the Food and Drug Administration (FDA, 2016). The major component of PEO is eugenol (>80%) (Suzuki et al., 2014). The PEO possesses antimicrobial activity against several microorganisms such as *Staphylococcus epidermidis, Proteus hauseri, Micrococcus yunnanensis*, and *Corynebacterium xerosis* (Suzuki et al., 2014). PEO also possesses *in vitro* antimicrobial activity against *Listeria monocytogenes* (Aureli et al., 1992). However, no studies have been conducted to explore the efficacy of PEO against *Salmonella* in poultry or poultry products. Therefore, the current study evaluated the efficacy of PEO and its nanoemulsion (PNE) on *S.* Heidelberg attachment on turkey skin. The specific objectives of the study were (1) to investigate the effect of PEO or PNE on reducing *S.* Heidelberg attachment (high inoculum and low inoculum) to turkey skin at 4 or 65°C, (2) to determine the effect of PEO or PNE on reducing *S.* Heidelberg (low inoculum) survival on skin during storage for 2 days at 4 or 10°C, and (3) to illustrate the effect of PEO on *S*. Heidelberg (low inoculum) attachment to the turkey skin using confocal microscopy (CM) and scanning electron microscopy (SEM).

## Materials and Methods

All biosafety procedures in Dr. Kollanoor Johny’s laboratory are approved by the Institutional Biosafety Committee at the University of Minnesota.

### Bacterial Strain and Growth Conditions

An MDR *S.* Heidelberg isolate from the 2011 ground turkey outbreak in the United States (kindly donated by Dr. Irene Hanning, College of Genome Sciences and Technology, University of Tennessee and Dr. Venkitanarayanan, University of Connecticut), was used for the study. Working cultures of *S.* Heidelberg was prepared from the glycerol stock cultures stored at -80°C. *S.* Heidelberg was made resistant to 50 μg/ml nalidixic acid sodium salt (NA; CAS. no. 3374-05-8, Alfa Aesar, Haverhill, MA, United States) for selective enumeration. The growth of *S.* Heidelberg (overnight culture) in tryptic soy broth (TSB; catalog no.C7141, Criterion, Hardy Diagnostics, Santa Maria, CA, United States) was determined on xylose lysine desoxycholate agar plates (XLD; catalog no. C 7322, Criterion, Hardy Diagnostics, Santa Maria, CA, United States) containing, 50 μg/ml NA and incubating at 37°C for 24 h. Then, final inoculum levels of 4 and 7 log_10_ CFU/ml were prepared from overnight broth culture (∼9 log_10_ CFU/ml) after centrifugation (3,600 × *g*, 15 min, 4°C) (Allegra X-14 R centrifuge, Beckman Coulter; 5350 Lakeview Parkway S Drive, Indianapolis, IN, United States) and suspending the pellets in sterile phosphate-buffered saline (PBS; pH 7.2) (Kollanoor-Johny et al., 2012a).

### Turkey Skin Preparation

Turkey drumsticks purchased from a local retail store were used for the study. The skin was separated from underlying muscles using a sterile scalpel, and 1 inch × 1 inch skin portions were exposed to UV light for 5 min to kill the background microbial flora before the application of *S.* Heidelberg on the skin surface (Kim et al., 1996).

### Inoculation of *S.* Heidelberg on Turkey Skin Surface

The skin samples were dipped in 100 ml PBS containing 4 and 7 log_10_ CFU/ml *S.* Heidelberg for 20 min. Skin samples were stored at room temperature in a biosafety cabinet for 1 h for *Salmonella* attachment. Unattached and loose *Salmonella* were removed by immersing the skin samples in sterile PBS for 5 min. Non-inoculated skin samples dipped in sterile PBS were used as negative controls (Kim et al., 1996; Tamblyn and Conner, 1997; Tamblyn et al., 1997).

### PEO, PNE, and Determination of Particle Size of PNE

PEO (≥99%; Natural, Food Grade; PCcode: 1002115007; Product: W290106-100G-K; Lot# MKBS7421V) was purchased from Sigma–Aldrich (St. Louis, MO, United States). PEO was mixed (by vortexing) in DI water for 30 s to prepare desired concentrations (0.5 and 1.0% v/v) for treating the turkey skin in all the experiments. PNE was prepared using high energy sonication technique as described previously (Ghosh et al., 2013; Bhargava et al., 2015). Briefly, PEO was emulsified in DI water adding tween 80 (2:1) and homogenized under high energy sonication (750 W) using a sonicator (Soniprep 450). The procedure was continued for 20 min with short intervals in an ice containing chamber to reduce the heat generation during sonication and to avoid the evaporative loss of the oil. The stability and particle size of PNE were determined using dynamic light scattering method after storing PNE at room temperature for 7 days (Bhargava et al., 2015).

### Dip Treatment of PEO or PNE on *S.* Heidelberg Attached to Turkey Skin at 65°C

PEO or PNE at concentrations of 0.5 and 1.0% (v/v) were freshly prepared in sterile DI water by vortexing for 30 s, and used in the study by maintaining the temperature at 65°C using a hot water bath (Model: 89032-226, VWR international, 1310 Goshen parkway, PA, United States). The temperature of the treatment water was set at 65°C to simulate the scalding step in poultry processing, and the temperature was monitored using a thermocouple for constancy. Each skin sample was inoculated with *S.* Heidelberg (4 log_10_ CFU/sq. inch or 7 log_10_ CFU/sq. inch) and separately dipped in 0, 0.5 or 1% (v/v) of PEO or PNE for the 30 s, 3 min, or 5 min. The skin samples were homogenized immediately after the dip treatment in fresh 10 ml PBS, and *S.* Heidelberg survival rate was determined. An industry control (chlorine, 50 ppm) and a solvent control (tween 80, 1%) were also tested with the lower inoculum level of *S*. Heidelberg at 65°C following the same protocol as detailed above to determine if these treatments had any effect on the pathogen populations.

### Dip Treatment of PEO or PNE on Skin Attached *S.* Heidelberg at Chilling Temperature

Concentrations of PEO or PNE at 0.5 and 1.0% (v/v) were freshly prepared in DI water by vortexing for 30 s, and used in the study by maintaining the temperature at 4°C. The temperature of the treatment water was maintained at 4°C to simulate chilling in poultry processing. Each skin sample was inoculated with *S.* Heidelberg (4 log_10_ CFU/sq. inch or 7 log_10_ CFU/sq. inch) and separately dipped in 0, 0.5, or 1% (v/v) of PEO or PNE for the 30 s, 3 min, or 5 min. Then skin samples were homogenized immediately after the dip treatment in fresh 10 ml PBS, and *S.* Heidelberg attachment was determined. An industry control (chlorine, 50 ppm) and a solvent control (tween 80, 1%) were also tested with the lower inoculum level of *S*. Heidelberg at 4°C following the same protocol as detailed above to determine if these treatments had any effect on the pathogen populations.

### Dip Treatment of PEO or PNE on *S*. Heidelberg Survival on Skin Surface during Storage at Chilling and Abuse Temperature

The PEO or PNE treatments at 1% (v/v) was prepared in sterile DI water by vortexing for 30 s, at 4°C and used for dip treating skin samples; *S.* Heidelberg inoculated (4 log_10_ CFU/sq. inch) and non-inoculated (control) skin samples for 5 min under chilling temperature were kept. Then the (non-dripping) samples were packaged under aerobic conditions and stored at 4 or 10°C for 48 h (FSIS, 2002; Ingham et al., 2007). *S.* Heidelberg attachment was determined by sampling at 0, 2, 24, and 48 h of storage after PEO and PNE treatments.

### Microbiological Analysis

The surviving *S.* Heidelberg attached on the skin after PEO or PNE treatments were determined by enumeration. The skin samples were homogenized with 10 ml sterile PBS in a stomacher (100/125V, 50/60Hz; Neutec Group Inc., 200 Central Ave, Farmingdale, NY, United States) for 2 min at 200 rpm. Then the sample homogenates were serially diluted 10-folds, and a volume of 100 μl from appropriate dilutions was surface plated on XLD containing 50 μg/ml NA. The surviving *S.* Heidelberg were enumerated after 24 h incubation at 37°C (Kollanoor-Johny et al., 2012a). A 100 μl of the dipping solution after all the treatments were directly surface plated on XLD + NA plates to determine any surviving pathogen populations in the dipping solution. Further, 1 ml of the dipping solution was enriched in 10 ml of selenite cysteine broth (Criterion, Hardy Diagnostics, Santa Maria, CA, United States), incubated for 12 h and streaked on XLD + NA plates for detection of surviving bacteria if any.

### CM of Turkey Skin Treated with PEO

Turkey skin (1 inch × 1 inch) portions were inoculated with 4 log_10_ CFU/sq. inch *S*. Heidelberg. Skin samples were dip treated in 1% PEO for 5 min at 4°C. Immediately after treatments, the skin samples were stained with L7012 LIVE/DEAD^®^ BacLight Bacterial Viability Kit (Catalog number: L7012, Thermo Fisher Scientific, Waltham, MA, United States) as described previously (Seo and Frank, 1999). Briefly, the inoculated and non-inoculated skin samples with or without treatment with PEO were immersed in a solution containing equal volume of SYTO^®^ 9 green-fluorescent nucleic acid stain (Catalog number: L7012, Thermo Fisher Scientific, Waltham, MA, United States) and propidium iodide Catalog number: L7012, Thermo Fisher Scientific, Waltham, MA, United States) red-fluorescent nucleic acid stain. The SYTO^®^ 9 dye stains both live and dead cells. However, propidium iodide penetrates only through damaged cell membranes (dead bacteria) and masks the intensity of SYTO^®^ 9 dye when both dyes are present. Therefore, live and dead bacteria appear as green and red, respectively. The skin samples were examined for live and dead bacteria under a confocal microscope (Nikon A1 spectral confocal microscope, University of Minnesota Imaging Center) after incubating at room temperature for 15 min. Non-inoculated skin samples dipped in sterile DI water or PEO served as negative controls. Similarly, *S*. Heidelberg inoculated skin samples dipped in sterile DI water for 5 min were included as positive controls.

### SEM of Turkey Skin Treated with PEO

Turkey skin samples (1 inch × 1 inch) inoculated with *S*. Heidelberg (4 log_10_ CFU/sq. inch) was dipped in 1% PEO for 5 min at 4°C. Non-inoculated skin samples dipped in sterile DI water or PEOs were used as negative controls. *S.* Heidelberg dipped in sterile DI water for 5 min served as *S*. Heidelberg controls. Sample preparation for electron microscopy was conducted as previously described with some modifications (Lee et al., 2014). Briefly, immediately after each treatment, the skin samples were stored in primary fixative [3% paraformaldehyde, 1.5% glutaraldehyde, and 2.5% sucrose in 0.1 M sodium cacodylate buffer with 5mM calcium chloride and 5mM magnesium chloride (pH 7.4)] for 12 h. Then the samples were fixed in 2% osmium tetroxide and 0.1 M sodium cacodylate buffer for 12 h. Then the samples were washed in ultrapure water (NANOpure Infinity^®^; Barnstead/Thermo Fisher Scientific; Waltham, MD, United States) and dehydrated by ascending grades of ethanol series. Samples were processed in a critical point dryer (Autosamdri-814; Tousimis; Rockville, MD, United States) and mounted on aluminum stubs, sputter-coated with gold-palladium, and observed using a scanning electron microscope (S3500N; Hitachi High Technologies America, Inc.; Schaumberg, Illinois; University of Minnesota Imaging Center) at an accelerating voltage of 5.00 kV.

### Statistical Analysis

A completely randomized design was used to analyze the effect of PEO and PNE treatments on *S*. Heidelberg in all experiments. Each skin sample served as the experimental unit. The number of *S.* Heidelberg colonies were logarithmically transformed before analysis to achieve homogeneity of variance (Byrd et al., 2003). The detection limit of *S.* Heidelberg was set at 1.0 log_10_ CFU/ml on XLD plates. The samples from which no bacteria were recovered after spread plating, but positive after enrichment, were assumed a value of 0.95 for analysis (9 CFU/ml) (Seo et al., 2000; Young et al., 2007). The data were analyzed using the PROC MIXED procedure of SAS (version 9.3) (SAS Institute, 2004). Differences among the least square means were detected using Fisher’s least significant difference test. A P value of <0.05 was considered statistically significant.

## Results and Discussion

### Effect of PEO or PNE against *S.* Heidelberg Attachment on Turkey Skin at Scalding Temperature (65°C)

Scalding is the step where the feather follicle is loosened for feather removal (picking) (Carrasco et al., 2012; FSIS, 2015). Scalding tanks are a potential area for carcass cross-contamination with pathogens such as *Salmonella* and *Campylobacter* (FSIS, 2015). Cross-contamination can lead to the attachment of *Salmonella* to the skin surface eventually making it hard to detach the pathogens with commonly used antimicrobial agents (Lillard, 1990; Kim et al., 1996; Yang et al., 2001; Nchez et al., 2002; Nagel et al., 2013).

**FIGURE 1:**
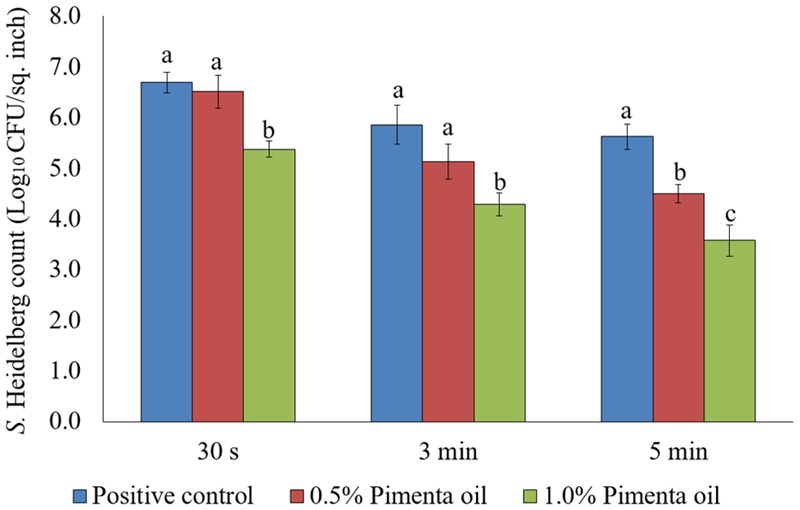
Effect of PEO against *S.* Heidelberg attachment on turkey skin at scalding temperature (65°C) at higher initial inoculum. *S.* Heidelberg (7 log_10_ CFU/sq. inch) inoculated skin samples were treated with 0, 0.5, or 1.0% PEO for 30 s, 3 min, or 5 min at 65°C. The skin samples were homogenized immediately after the dip treatment and the pathogen populations were determined. The treatments were significantly different at *P* < 0.05.

**FIGURE 2:**
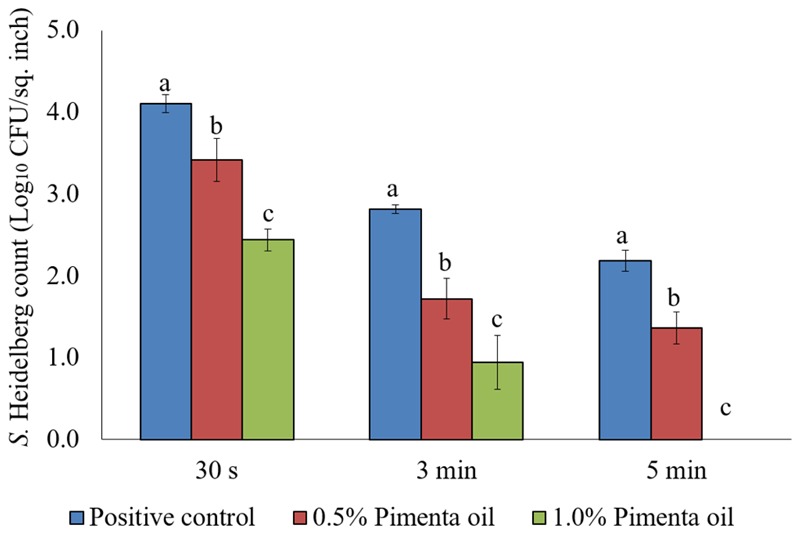
Effect of PEO against *S.* Heidelberg attachment on turkey skin at scalding temperature (65°C) at lower initial inoculum. *S.* Heidelberg (4 log_10_ CFU/sq. inch) inoculated skin samples were treated with 0, 0.5, or 1.0% PEO for 30 s, 3 min, or 5 min at 65°C. The skin samples were homogenized immediately after the dip treatment and the pathogen populations were determined. The treatments were significantly different at *P* < 0.05.

In the current study, two levels of initial inocula were tested: a higher inoculum level of 7 log_10_ CFU/sq. inch, and a lower inoculation level of 4 log_10_ CFU/sq. inch. The application of PEO resulted in a significant reduction of *S.* Heidelberg attachment on turkey skin at scalding temperature when a high-level inoculum of *S.* Heidelberg was used (**Figure 1**). The PEO treatment at a concentration of 0.5% resulted in 1.13 log_10_ CFU/sq. inch reduction of *S.* Heidelberg after 5 min dip treatment (*P* < 0.05) at 65°C, compared to the *Salmonella* control. Whereas, 1% PEO dip treatment resulted in 1.37, 1.57 and 2.05 log_10_ CFU/sq. inch reduction of *S.* Heidelberg in 30 s, 3 min, and 5 min, respectively at 65°C (*P* < 0.05) (**Figure 1**). The PEO treatment was also effective when a low initial inoculum of *S.* Heidelberg was tested and was concentration dependent (**Figure 2**). The PEO treatment at the lower concentration of 0.5% resulted in 0.72, 1.09, and 1.87 log_10_ CFU/sq. inch reduction of *S.* Heidelberg in 30 s, 3 min, and 5 min dip treatment, respectively at 65°C (*P* < 0.05) whereas, 1% PEO dip treatment resulted in 1.66, 1.87, and 2.19 log_10_ CFU/sq. inch reduction of *S.* Heidelberg in 30 s, 3 min, and 5 min (enrichment negative), respectively 65°C (*P* < 0.05) (**Figure 2**).

Chlorine is a common antimicrobial agent used in poultry processing (Sohaib et al., 2015). However, chlorine may lose its efficacy in scalding tanks due to the high organic load associated with the carcass, and the evaporative loss of the chlorine over time in the scalding tanks (FSIS, 2015). Organic acids can be used in scalding tanks. However, organic acids such as acetic, citric, lactic, malic, mandelic, propionic, or tartaric acid require a concentration of 2.0–6.0% to obtain 2 log_10_ CFU or more reduction of *Salmonella* attached to the skin of broiler carcasses (Tamblyn and Conner, 1997). In addition, previous studies reported the reduced activity of commonly used synthetic antimicrobial agents such as sodium hypochlorite, acetic acid, trisodium phosphate, and sodium metabisulfite against *Salmonella* attached to poultry skin, and revealed the potential of increased resistance of *Salmonella* to these antimicrobial treatments (Tamblyn et al., 1997). In the current study, *S.* Heidelberg reduction obtained with the use of 1% PEO after 5 min at the lower inoculum level tested was comparable to that of organic acids, and other USDA approved synthetic antimicrobial agents (Tamblyn et al., 1997; Tamblyn and Conner, 1997).

**FIGURE 3:**
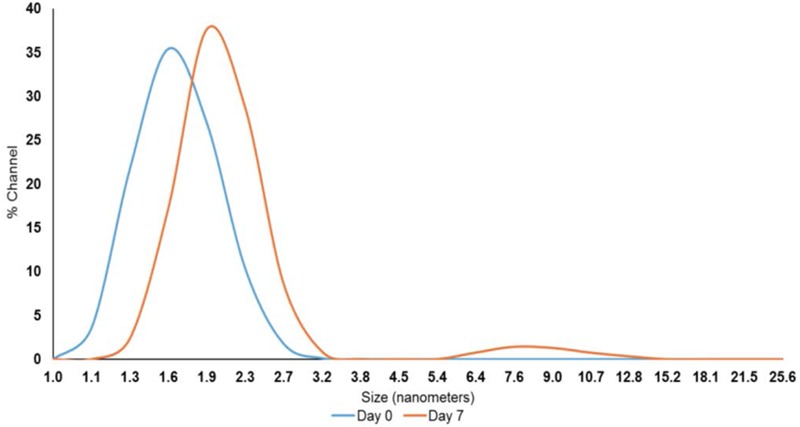
Pimenta nanoemulsion (PNE) was prepared by emulsifying PEO in DI water using tween 80 (2:1) and homogenized under high energy sonication (750 W) using a sonicator. The procedure was continued for 20 min with short intervals. The stability and particle size of PNE were determined using dynamic light scattering method after storing PNE at room temperature for 7 days.

After exploring the efficacy of PEO at both inoculum levels, we focused on the lower inoculum (4 log_10_ CFU/sq. inch) to test the effect of PNE. Although the reported contamination level of *Salmonella* on poultry carcasses exiting the chillers is ∼ 2.0 log_10_ CFU/carcass (Waldroup, 1996), we increased the inoculum to 4 log_10_ CFU/sq. inch to avoid any potential bias in determining the efficacy of PNE treatment. The nanoemulsion of PEO (PNE) could increase the solubility and stability of PEO in water, and the fine droplet size could increase the availability of PEO (Landry et al., 2014). The peak droplet size of the prepared PNE remained in the range of 1.6–2.3 nm during a storage period of 7 days at room temperature which could indicate its increased stability in the water (**Figure 3**).

**FIGURE 4:**
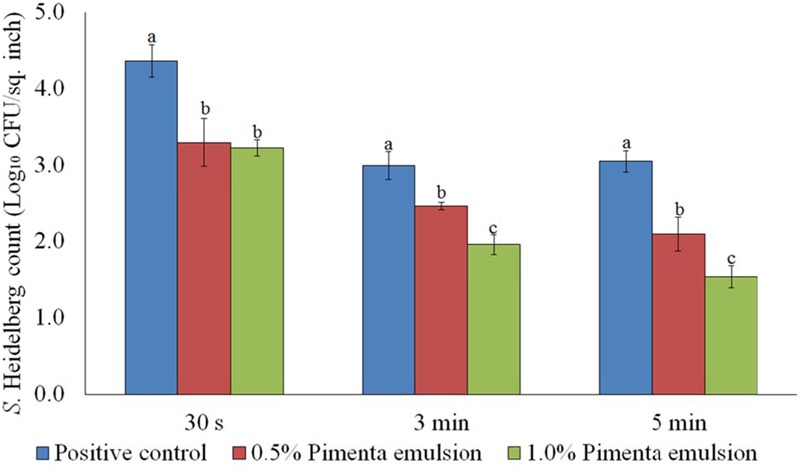
Effect of PNE against *S.* Heidelberg attachment on turkey skin at scalding temperature (65°C) for lower initial inoculum. *S.* Heidelberg (4 log_10_ CFU/sq. inch) inoculated skin samples were treated with 0, 0.5, or 1.0% PNE for 30 s, 3 min, or 5 min at 65°C. The skin samples were homogenized immediately after the dip treatment and the pathogen populations were determined. The treatments were significantly different at *P* < 0.05.

The PNE treatment resulted in significant reduction of *S.* Heidelberg on the skin which was comparable to the respective concentrations of PEO at 65°C. The PNE treatment at 0.5% resulted in 1.07, 0.53 and 0.95 log_10_ CFU/sq. inch reduction of skin-attached *S.* Heidelberg after 30 s, 3 min, and 5 min dip treatment, respectively at 65°C (*P* < 0.05). Similarly, 1.0% PNE resulted in 1.14, 1.03, and 1.51 log_10_ CFU/sq. inch reductions of *S.* Heidelberg after 30 s, 3 min, and 5 min dip treatment, respectively at 65°C (*P* < 0.05; **Figure 4**). In the current experiment, both PEO and PNE showed similar efficacy since PEO also had small droplet size and uniform distribution on the skin surface due to the vigorous homogenization in DI water before application on the skin surface.

**FIGURE 5:**
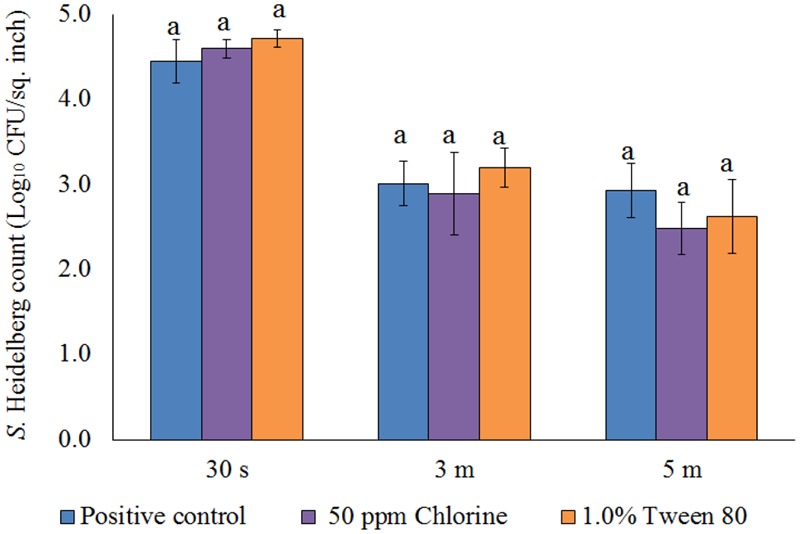
Effect of chlorine (50 ppm) and tween 80 (1.0%) against *S.* Heidelberg attachment on turkey skin at scalding temperature (65°C) for lower initial inoculum. *S.* Heidelberg (4 log_10_ CFU/sq. inch) inoculated skin samples were treated with 50 ppm chlorine or 1.0% tween 80 for 30 s, 3 min, or 5 min at 65°C. The skin samples were homogenized immediately after the dip treatment and the pathogen populations were determined.

After the treatments, no *S*. Heidelberg was detected in the dipping solution containing PNE and PEO treatments (enrichment negative). However, a substantial population of the pathogen survived in the control dipping solution. The positive controls had 2.2-, 1.9-, and 2.3- log_10_ CFU/ml of *S*. Heidelberg after 30 s, 3 min, and 5 min dip treatments at 65°C. In addition, we conducted experiments using chlorine (50 ppm; industry control) and tween 80 (1.0%; solvent control) and found that these controls had no effect on the skin attached *S.* Heidelberg at 65°C compared to the *Salmonella* control (*P* > 0.05; **Figure 5**).

### Effect of PEO or PNE against *S.* Heidelberg Attachment on Turkey Skin at Chilling Temperature (4°C)

**FIGURE 6:**
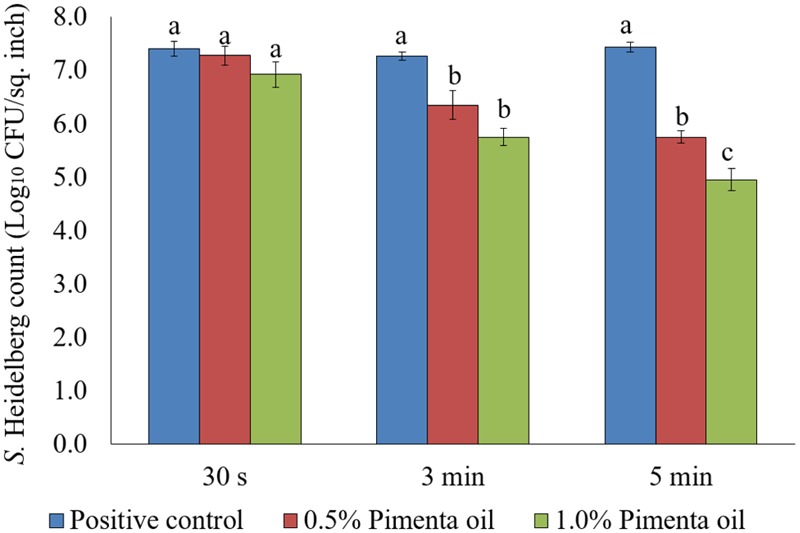
Effect of PEO against *S.* Heidelberg attachment on turkey skin at chilling temperature (4°C) for higher initial inoculum. *S.* Heidelberg (7 log_10_ CFU/sq. inch) inoculated skin samples were treated with 0, 0.5, or 1.0% PEO for 30 s, 3 min, or 5 min at 4°C. The skin samples were homogenized immediately after the dip treatment and the pathogen populations were determined. The treatments were significantly different at *P* < 0.05.

Chilling is a critical step in poultry processing where the internal temperature of the poultry carcass is reduced to 40°F (4.4°C) or below within 4–8 h of slaughtering to prevent the growth of pathogenic bacteria including *Salmonella* (FSIS, 2014). The cross-contamination of carcasses with *Salmonella* is high in chiller where the carcasses are immersed in chilling water, and the carcass exiting the chiller tank often carry a significant amount of the pathogen (Lillard, 1990; Nchez et al., 2002). In the current study, the PEO application as dip treatment at 4°C significantly reduced the attachment of *S.* Heidelberg on turkey skin and the reduction was concentration and contact time dependent after 5 min of exposure for higher inoculation (**Figure 6**). Dip treatment of PEO at 0.5 and 1% did not result in significant reduction of skin attached *S.* Heidelberg for a contact period of 30 s at 4°C when higher initial inoculum of *S.* Heidelberg was used (*P* > 0.05; **Figure 6**). However, 3- and 5- min dip treatment with PEO resulted in a significant reduction of *S.* Heidelberg compared to the control at both concentrations tested (*P* < 0.05; **Figure 6**).

**FIGURE 7:**
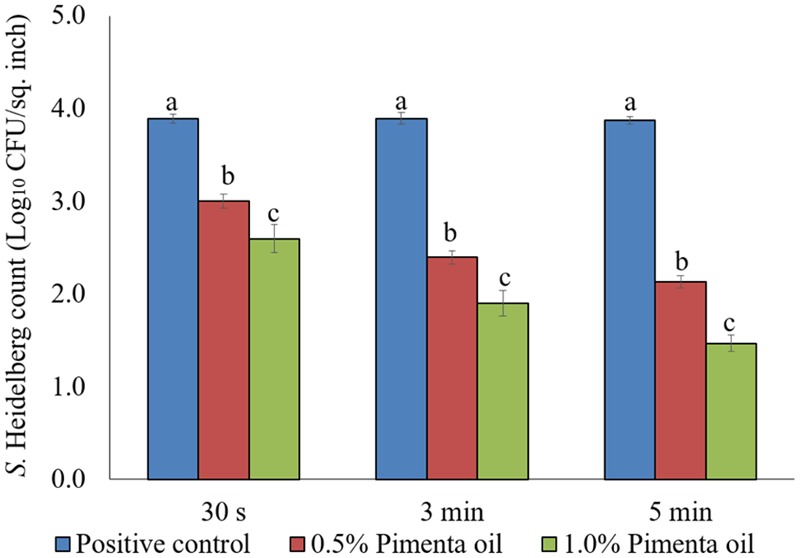
Effect of PEO against *S.* Heidelberg attachment on turkey skin at chilling temperature (4°C) for lower initial inoculum. *S.* Heidelberg (4 log_10_ CFU/sq. inch) inoculated skin samples were treated with 0, 0.5, or 1.0% PEO for 30 s, 3 min, or 5 min at 4°C. The skin samples were homogenized immediately after the dip treatment and the pathogen populations were determined. The treatments were significantly different at *P* < 0.05.

The PEO treatment showed higher efficacy against *S.* Heidelberg at 4°C with the low initial inoculum of *Salmonella* (4 log_10_ CFU/sq. inch skin) compared to the higher inoculation (7 log_10_ CFU/sq. inch skin). In addition, both PEO concentrations resulted in rapid reduction of *S.* Heidelberg for a contact time as low as 30 s (*P* < 0.05; **Figure 7**). For the 0.5% PEO dip treatment, reduction of 0.89, 1.50, and 1.74 log_10_ CFU/sq. inch was obtained with 30 s, 3 min, and 5 min contact time, respectively at 4°C, compared to the controls (*P* < 0.05), whereas, a reduction of 1.3, 1.99, and 2.40 log_10_ CFU/sq. inch was obtained with 1.0% PEO after 30 s, 3 min, and 5 min dip treatment, compared to the control at 4°C (*P* < 0.05; **Figure 7**).

In the current study, PEO was rapidly effective against *S.* Heidelberg attachment on turkey skin (3 or 5 min for higher initial inoculum, and 30 s, 3 min, or 5 min for lower initial inoculum). As a usual practice, poultry carcass is immersed in the chilling tank at 4.4°C, and the antimicrobial agents get sufficient contact time to reduce *Salmonella* (Nagel et al., 2013). However, common antimicrobial agents including chlorine were found to be less effective against skin-attached *Salmonella*. Chlorine reduced less than 1.0 log_10_ CFU/ml of *S.* Typhimurium attachment on poultry skin at a concentration of 50 ppm for 50 min contact time (Yang et al., 2001). In our study, the PEO dip treatment showed similar or better/higher efficacy compared to other synthetic antimicrobial agents approved for the chilling process. For example, a common antimicrobial, sodium metabisulfite, was ineffective against firmly attached *Salmonella* even after 60 min immersion treatment at 0°C. In addition, acetic acid (5%) and sodium metabisulfite (1%) were ineffective against *S.* Typhimurium attachment on poultry skin under aforementioned chilling conditions (Tamblyn et al., 1997). Additionally, organic acids such as acetic, citric, lactic, malic, mandelic, propionic, or tartaric acid required a concentration of 4.0% or above to get at least 2 log_10_ reduction of *S.* Typhimurium attachment on poultry skin at 0°C for 60 min contact time (Tamblyn and Conner, 1997). Another antimicrobial, sodium hypochlorite, was effective against skin-attached *S.* Typhimurium only when used at higher concentrations such as 400 and 800 ppm for a contact time of 60 min at 0°C to result in <2 log_10_ CFU/ml reductions of firmly attached *Salmonella* on the skin surface.

In addition to the potential use of PEO in chilling tanks, it could be used for post-chill dip treatment during poultry processing since PEO dip treatment was effective rapidly against skin-attached *S*. Heidelberg. Post-chill dip application of antimicrobial agents is currently practiced as a part of the multiple hurdle technology along with antimicrobial interventions in the chilling tanks (Nagel et al., 2013; Nair et al., 2015). The advantage of post-chill dip application is that the carcass is in contact with the antimicrobial agent for a shorter duration (30 s) and a higher concentration of antimicrobial agent can be used without deteriorating the carcass quality. The antimicrobial agent would be more efficient since there is less organic load compared to the chilling tank (Nagel et al., 2013). In the current study, 30 s dip treatment of PEO resulted in 0.83 and 1.3 log_10_ CFU/sq. inch reductions of *S.* Heidelberg with 0.5 and 1.0% PEO, respectively at 4°C. It is comparable to the reduction achieved during post chill dip treatment using other synthetic antimicrobial agents or other EOs (Nagel et al., 2013; Nair et al., 2014, 2015). For example, 40 or 400 ppm chlorine and 1000 or 5000 ppm lysozyme resulted in less than 1 log_10_ CFU/ml reduction of *S.* Typhimurium on broiler carcass when used as post-chill dip treatment for 20 s (Nagel et al., 2013). Similarly, post chill application of carvacrol at 0.5, 1.0, or 2% for 30 s resulted in less than a log_10_ CFU/ml reduction of *Salmonella* spp. (*S.* Enteritidis, *S.* Heidelberg, and *S.* Typhimurium) on skinless, boneless turkey breast cutlets (Nair et al., 2014).

**FIGURE 8:**
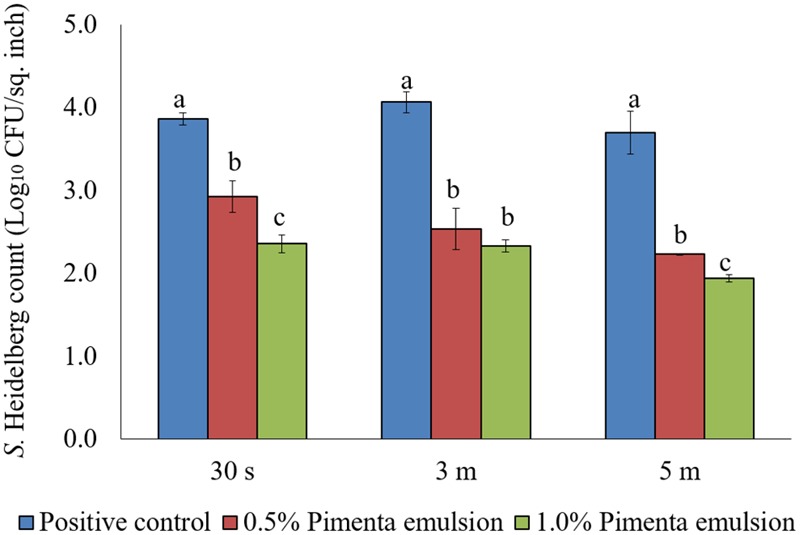
Effect of PNE against *S.* Heidelberg attachment on turkey skin at chilling temperature (4°C) for lower initial inoculum. *S.* Heidelberg (4 log_10_ CFU/sq. inch) inoculated skin samples were treated with 0, 0.5, or 1.0% PNE for 30 s, 3 min, or 5 min at 4°C. The skin samples were homogenized immediately after the dip treatment and the pathogen populations were determined. The treatments were significantly different at *P* < 0.05.

The PNE treatment also showed significant reduction of skin-attached *S.* Heidelberg at 4°C (**Figure 8**). Lower initial inoculum (4.0 log_10_ CFU/sq. inch) of *S.* Heidelberg was used to study the effect of PNE on skin-attached *S.* Heidelberg since PEO treatment showed similar or higher efficacy when used against the lower initial inoculum of *S.* Heidelberg. At chilling temperature, PNE at 0.5% resulted in 0.94, 1.53 and 1.47 log_10_ CFU/sq. inch reductions of *S.* Heidelberg in 30 s, 3 min, and 5 min dip treatment, respectively (*P* < 0.05), compared to the *S.* Heidelberg control. Likewise, PNE at 1% resulted in 1.51, 1.74, and 1.76 log_10_ CFU/sq. inch reductions of *S.* Heidelberg after 30 s, 3 min, and 5 min dip treatment, respectively at 4°C compared to the control (*P* < 0.05) (**Figure 8**).

**FIGURE 9:**
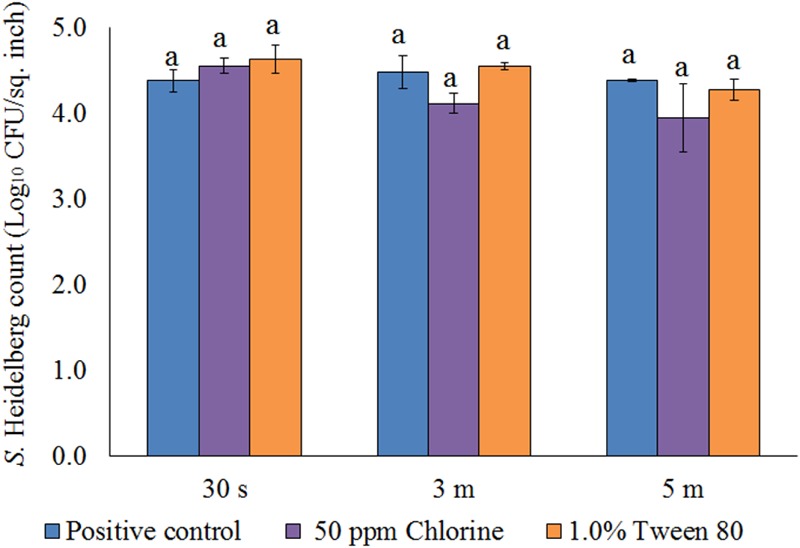
Effect of chlorine (50 ppm) and tween 80 (1.0%) against *S.* Heidelberg attachment on turkey skin at chilling temperature (4°C) for lower initial inoculum. *S.* Heidelberg (4 log_10_ CFU/sq. inch) inoculated skin samples were treated with 50 ppm chlorine or 1.0% tween 80 for 30 s, 3 min, or 5 min at 65°C. The skin samples were homogenized immediately after the dip treatment and the pathogen populations were determined.

After the treatments, no *S*. Heidelberg was detected in the dipping solution containing PNE and PEO treatments (enrichment negative). However, a substantial population of the pathogen survived in the control dipping solution. The positive controls had 2.5-, 2.4-, and 2.6- log_10_ CFU/ml of *S*. Heidelberg after 30 s, 3 min and 5 min dip treatments at 4°C. In addition, we conducted experiments using chlorine (50 ppm; industry control) and tween 80 (1.0%; solvent control) and found that these controls had no effect on the skin attached *S.* Heidelberg at 4°C compared to the *Salmonella* control. Chlorine treatment resulted in only 0.37 and 0.44 log_10_ CFU/sq. inch reductions of *S.* Heidelberg (*P* > 0.05; **Figure 9**) for 3 and 5 min dip treatments, respectively whereas tween 80 treatment resulted in same *S.* Heidelberg count as that of *Salmonella* control in all the three dip treatments at 4°C (*P* > 0.05; **Figure 9**).

### Effect of PEO or PNE against *S.* Heidelberg Attachment on Turkey Skin at 4°C and Stored for 48 h at 4°C and 10°C

**FIGURE 10:**
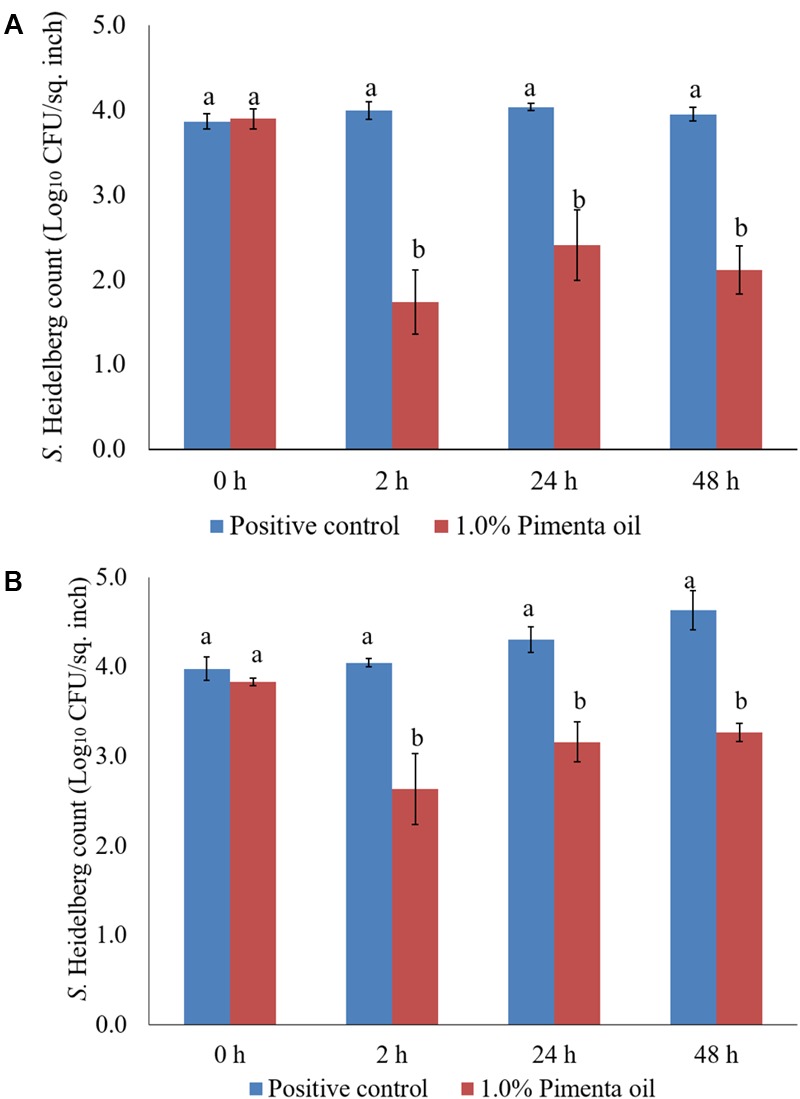
**(A)** Effect of PEO against *S.* Heidelberg attachment on turkey skin at chilling temperature (4°C) and storage at 4°C for 48 h. *S.* Heidelberg (4 log_10_ CFU/sq. inch) inoculated skin samples were treated with 0 or 1.0% PEO for 5 min at 4°C. The samples were packaged under aerobic conditions and stored at 4°C. *S.* Heidelberg attachment was determined by sampling at 0, 2, 24, and 48 h of storage after PEO treatments. The treatments were significantly different at *P* < 0.05. **(B)** Effect of PEO against *S.* Heidelberg attachment on turkey skin at chilling temperature (4°C) and storage at 10°C for 48 h. *S.* Heidelberg (4 log_10_ CFU/sq. inch) inoculated skin samples were treated with 0 or 1.0% PEO for 5 min at 4°C. The samples were packaged under aerobic conditions and stored at 10°C. *S.* Heidelberg attachment was determined by sampling at 0, 2, 24, and 48 h of storage after PEO treatments. The treatments were significantly different at *P* < 0.05.

The PEO treatment at 1% applied for 5 min at 4°C showed a higher reduction of *S.* Heidelberg attachment on turkey skin (**Figure 7**). Therefore, the same combination was used for determining the efficacy of PEO or PNE treatments against *S.* Heidelberg attachment on turkey skin for 48 h storage at 4°C (**Figure 10A**) and 10°C (**Figure 10B**; abuse temperature). The PEO treatment (1.0%) resulted in 2.27, 1.63, and 1.83 log_10_ CFU/sq. inch reduction of *S.* Heidelberg after 2, 24 and 48 h of storage at 4°C (*P* < 0.05) (**Figure 10A**). The skin samples were also stored at 10°C to determine *S.* Heidelberg attachment at abuse temperature during refrigerated storage (FSIS, 2002). *S.* Heidelberg multiplied during 10°C storage in the *S*. Heidelberg controls. However, 1% PEO dip treatment for 5 min significantly reduced *S.* Heidelberg growth and multiplication on the skin after 2, 24, and 48 h of storage at 10°C (*P* < 0.05; **Figure 10B**).

**FIGURE 11:**
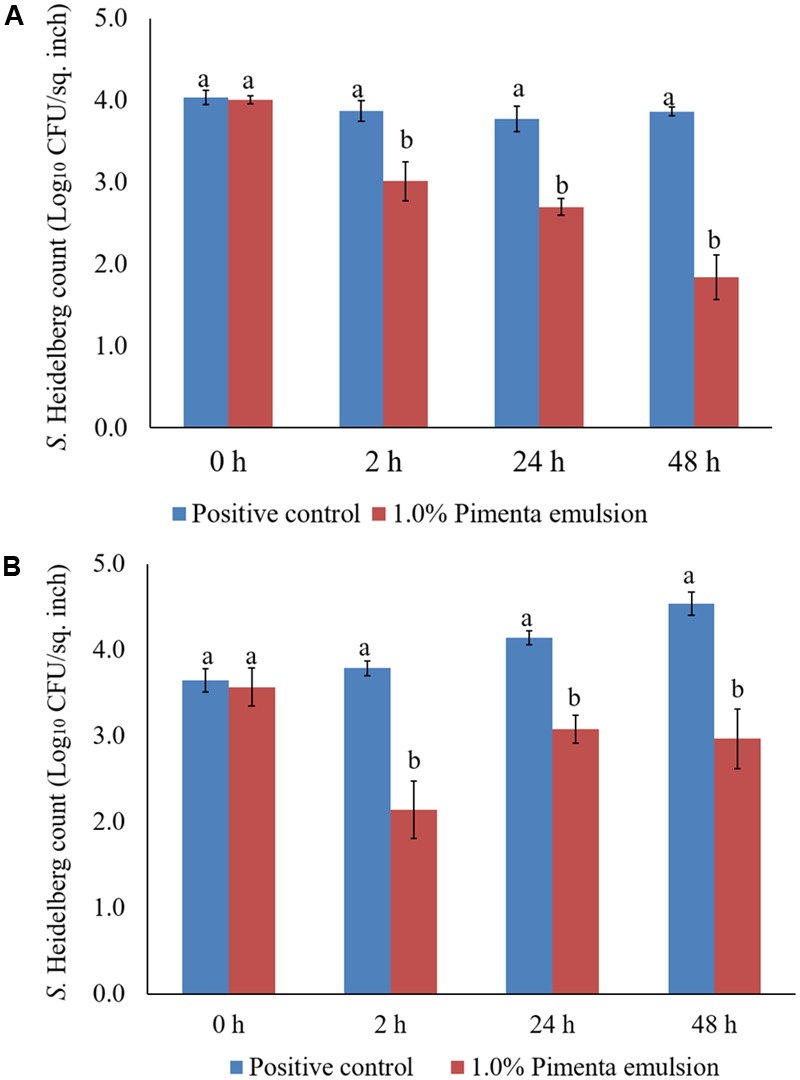
**(A)** Effect of PNE against *S.* Heidelberg attachment on turkey skin at chilling temperature (4°C) and storage at 4°C for 48 h. *S.* Heidelberg (4 log_10_ CFU/sq. inch) inoculated skin samples were treated with 0 or 1.0% PNE for 5 min at 4°C. The samples were packaged under aerobic conditions and stored at 4°C. *S.* Heidelberg attachment was determined by sampling at 0, 2, 24, and 48 h of storage after PEO treatments. The treatments were significantly different at *P* < 0.05. **(B)** Effect of PNE against *S.* Heidelberg attachment on turkey skin at chilling temperature (4°C) and storage at 10°C for 48 h. *S.* Heidelberg (4 log_10_ CFU/sq. inch) inoculated skin samples were treated with 0 or 1.0% PEO for 5 min at 4°C. The samples were packaged under aerobic conditions and stored at 10°C. *S.* Heidelberg attachment was determined by sampling at 0, 2, 24, and 48 h of storage after PNE treatments. The treatments were significantly different at *P* < 0.05.

The PNE (1.0%) treatment was also effective in reducing *S.* Heidelberg attachment on turkey skin at 4 and 10°C; the efficacy was comparable to the 1.0% PEO treatment for 5 min under similar storage conditions. The PNE treatment resulted in significant reduction in *S.* Heidelberg attachment after 2, 24 and 48 h of storage at 4°C (*P* < 0.05; **Figure 11A**). Likewise, PNE resulted in a similar reduction in *S.* Heidelberg populations after storage at 10°C (*P* < 0.05; **Figure 11B**). Therefore, the results of the present study indicate that PEO and PNE treatments are effective in reducing *S.* Heidelberg attachment on turkey skin when stored at 4°C for 48 h. Also, PEO and PNE treatments were effective in reducing *S.* Heidelberg attachment on turkey skin during abuse temperature (10°C) condition for 48 h.

### Effect of PEO on *S*. Heidelberg Attachment to Turkey Skin Illustrated Using CM and SEM

**FIGURE 12:**
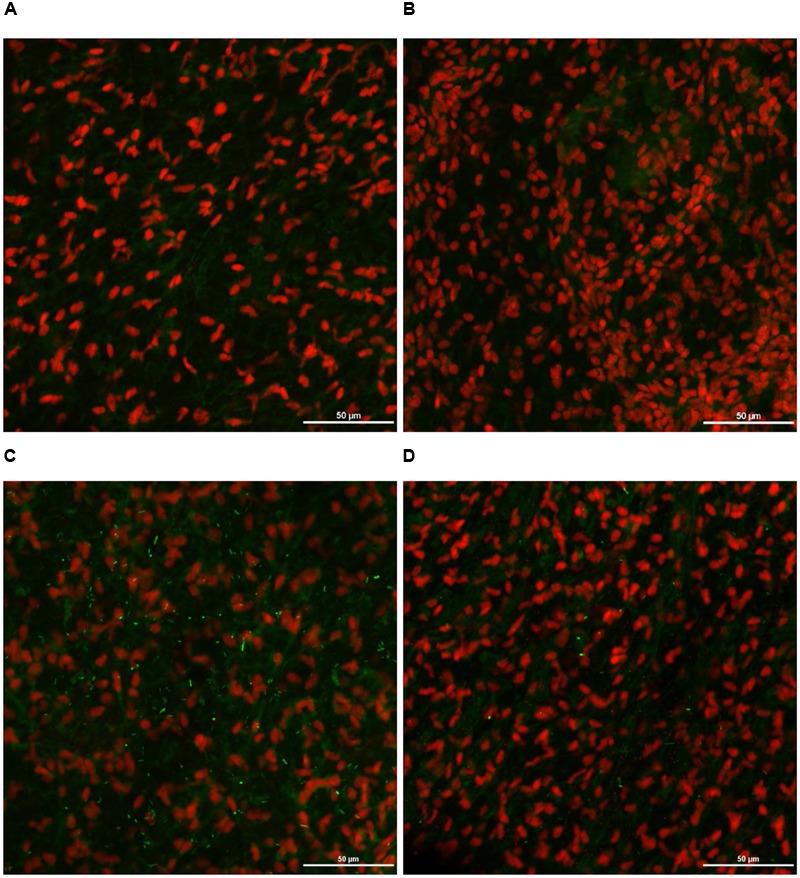
Effect of PEO against *S.* Heidelberg attachment on turkey skin at simulated chilling temperature (4°C) – Confocal microscopy of the outer surface of skin: **(A)** Negative control (Turkey skin + DI water), **(B)** Pimenta control (Turkey skin + DI water + PEO), **(C)** *Salmonella* control (Turkey skin + DI water + *S.* Heidelberg) and **(D)** Treatment (Turkey skin + DI water + *S.* Heidelberg + PEO).

**FIGURE 13:**
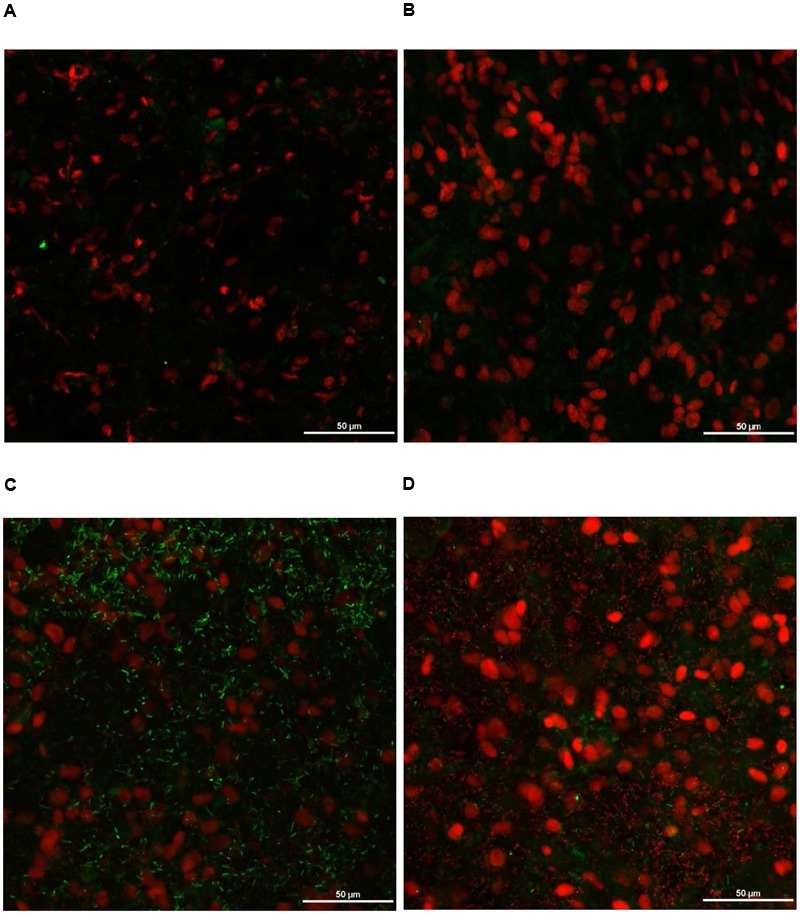
Effect of PEO against *S.* Heidelberg attachment on turkey skin at simulated chilling temperature (4°C) – Confocal microscopy of the inner surface of skin: **(A)** Negative control (Turkey skin + DI water), **(B)** Pimenta control (Turkey skin + DI water + PEO), **(C)** *Salmonella* control (Turkey skin + DI water + *S.* Heidelberg) and **(D)** Treatment (Turkey skin + DI water + *S.* Heidelberg + PEO).

**FIGURE 14:**
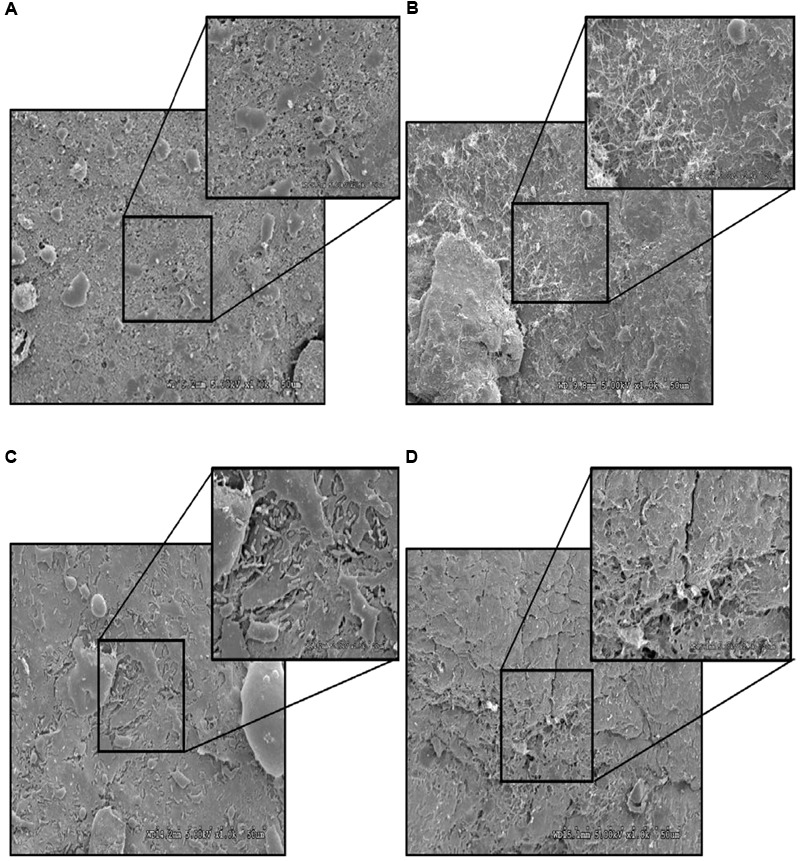
Effect of PEO against *S.* Heidelberg attachment on turkey skin at simulated chilling temperature (4°C) – Scanning electron microscopy of the outer surface of skin: **(A)** Negative control (Turkey skin + DI water), **(B)** Pimenta control (Turkey skin + DI water + PEO), **(C)** *Salmonella* control (Turkey skin + DI water + *S.* Heidelberg) and **(D)** Treatment (Turkey skin + DI water + *S.* Heidelberg + PEO).

**FIGURE 15:**
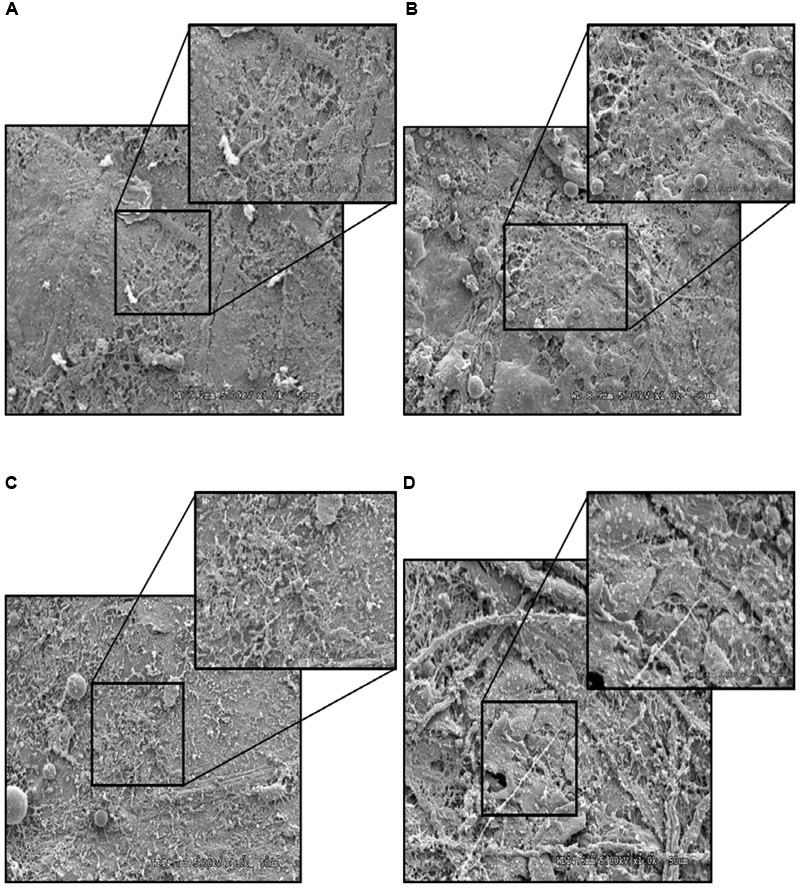
Effect of PEO against *S.* Heidelberg attachment on turkey skin at simulated chilling temperature (4°C) – Scanning electron microscopy of the inner surface of skin: **(A)** Negative control (Turkey skin + DI water), **(B)** Pimenta control (Turkey skin + DI water + PEO), **(C)** *Salmonella* control (Turkey skin + DI water + *S.* Heidelberg) and **(D)** Treatment (Turkey skin + DI water + *S.* Heidelberg + PEO).

As expected, *S*. Heidelberg was not present in the negative controls (**Figures 12A, 13A, 14A, 15A**). The CM and SEM revealed that the PEO treatment had no deleterious effect on turkey skin. The skin cells maintained normal shape (**Figures 12B, 13B, 14B, 15B**). Inoculation of skin samples with *S.* Heidelberg resulted in the attachment of the pathogen to both sides. The physical structure of the skin surface contributed to the firm attachment of *S.* Heidelberg onto the skin surface. The pathogen could penetrate deep into the crevices and feather follicles on the outer skin surface. In addition, the swelling of skin cells during the immersion process could have exposed deep channels and crevices on the skin resulting in enhanced *S.* Heidelberg attachment to the skin surface (Kim et al., 1996). The presence of flagella in *Salmonella* is also a major contributing factor for firm attachment of the pathogen to the poultry skin. Flagellar structural subunits (*flgK, fliC*, and *fljB*) and motor units (*motA, motB*) play a significant role in the *Salmonella* attachment to the poultry skin (Salehi et al., 2017).

The CM of the *S.* Heidelberg treated skin revealed many live *S.* Heidelberg cells (green rod-shaped cells) on the outer (**Figure 12C**) and inner (**Figure 13C**) surfaces of the skin. However, on the PEO treated skin, *S.* Heidelberg attachment was significantly less (**Figures 12D, 13D**) as indicated by several dead cells (red rod-shaped cells) (**Figure 13D**). Similarly, SEM also revealed a significant reduction of *S.* Heidelberg on the inner and outer surfaces of the skin in the PEO treated skin samples (**Figures 14D, 15D**) compared to the *S.* Heidelberg controls (**Figures 14C, 15C**). The attachment of *S.* Heidelberg was more on the inner surface of the skin relative to the outer surface which could be due to the strong adherence of *Salmonella* in the adipose tissue underneath the skin (Tan et al., 2014).

### Potential Mechanisms of Action of EOs against *S*. Heidelberg

The active components present in the EOs elicit antimicrobial property against pathogenic microorganisms (Kollanoor Johny et al., 2008; Amalaradjou et al., 2010; Kollanoor-Johny et al., 2012a,b; Nair et al., 2014, 2015). These components include eugenol, carvacrol, thymol, cinnamaldehyde, *p*-cymene and a multitude of others that kill microorganisms by different modes of actions such as disruption of the cell wall, degradation of cytoplasmic membranes, alteration of membrane potential, disruption of proton motive force, and leakage of cellular contents through multiple targets in the microbial cell (Burt, 2004). However, the mode of action of essential oils could vary between Gram-negative and Gram-positive bacteria. The features of Gram negative bacterial cell such as less lipophilic nature, the presence of outer membrane and the lipopolysaccharide could make Gram negative bacteria more resistant to the effect of essential oil components (Nazzaro et al., 2013). On the other side, we have previously reported high antibacterial activity for essential oil ingredients such as *trans*-cinnamaldehyde and eugenol against *S*. Enteritidis in broiler chickens (Kollanoor-Johny et al., 2012a,b).

In the present study, the PEO treatment caused significant reduction of *S.* Heidelberg attached to turkey skin. The exact mechanism of action of PEO against *S.* Heidelberg has not been understood. However, PEO contains eugenol as a major active component having strong inhibitory activity against both Gram-negative and Gram-positive bacteria (Hyldgaard et al., 2012). The presence of eugenol might have resulted in morphological alterations of the microbial cell, including the disruption of the cell membrane, and formation cleft, and pore leading to the leakage of contents and subsequent death of microorganisms (Suzuki et al., 2014). Moreover, eugenol can down-regulate several critical genes in *S*. Enteritidis responsible to adhesion and invasion of cultured avian epithelial cells *in vitro* (Kollanoor-Johny et al., 2012a; Upadhyaya et al., 2013). More investigations are required to understand the exact mechanism of action of PEO on *S*. Heidelberg attached to turkey skin.

## Conclusion

In the current study, we investigated the potential of PEO and PNE on *S*. Heidelberg attached to turkey skin in simulated scalding and chilling conditions. The highest reduction of skin attached *S.* Heidelberg was observed when 1.0% PEO or PNE was used for 5 min dip treatment at scalding (65°C) or chilling temperature (4°C). Although comparable *S*. Heidelberg reductions were obtained at both temperatures, a slightly better effect was noticed with 1% PEO at the tested scalding temperature. An antimicrobial treatment that results in 2 log or more reduction of *Salmonella* when applied during chilling is considered effective since *Salmonella* may be present on the poultry carcass in the range of <100 cells after processing (Tamblyn et al., 1997). Interestingly, no *S*. Heidelberg was detected in the dipping solution containing PNE and PEO treatments, although the pathogens were present in the control dipping solution. This result indicates that PEO and PNE could prevent cross-contamination or recontamination of the pathogens in case the same water is used for dipping/washing carcasses. Moreover, PEO and PNE were effective in reducing *S.* Heidelberg on skin during a storage period of 2 days at chilling and abuse temperatures. CM and SEM revealed a deep infiltration and attachment of *S.* Heidelberg in the inner and outer surfaces of the skin. Treatment with PEO reduced pathogen attachment on either side as evidenced from the CM and SEM images.

Overall, the results indicate that PEO or PNE treatments could be potential alternatives to synthetic antimicrobial agents to reduce *S*. Heidelberg attachment to turkey skin during poultry processing. Although the PEO was solubilized by the nanoemulsion method, the potency did not differ significantly between the PEO and PNE treatments. We are investigating the possibility of scale-up investigations with PEO by incorporating other parameters such as different *Salmonella enterica* serotypes of importance, exploring techniques to lower the levels of PNE and PEO for application on turkey carcasses, varying levels of organic content in the water, the age of scalding and chill water, and the effect of PEO during long-term storage. Furthermore, sensory evaluation of carcasses treated with PEO and PNE treatments must be carried out.

## Author Contributions

AKJ conceived the idea and designed the experiments. DN performed all experiments and participated in the statistical analysis with AKJ. DN and AKJ jointly wrote and corrected the manuscript.

## Conflict of Interest Statement

The authors declare that the research was conducted in the absence of any commercial or financial relationships that could be construed as a potential conflict of interest.

## References

[B1] Amalaradjou M. A. R. Baskaran S. A. Ramanathan R. Kollanoor Johny A. Charles A. S. Valipe S. R. (2010). Enhancing the thermal destruction of *Escherichia coli* O157: H7 in ground beef patties by trans-cinnamaldehyde. *Food Microbiol.* 27 841–844. 10.1016/j.fm.2010.05.006 20630328

[B2] Aureli P. Costantini A. Zolea S. (1992). Antibacterial activity of some plant essential oils against *Listeria monocytogenes*. *J. Food Prot.* 55 344–348. 10.4315/0362-028X-55.5.344 31071867

[B3] Bhargava K. Conti D. S. da Rocha S. R. P. Zhang Y. (2015). Application of an oregano oil nanoemulsion to the control of foodborne bacteria on fresh lettuce. *Food Microbiol.* 47 69–73. 10.1016/j.fm.2014.11.007 25583339

[B4] Burt S. (2004). Essential oils: their antibacterial properties and potential applications in foods—a review. *Int. J. Food Microbiol.* 94 223–253. 10.1016/j.ijfoodmicro.2004.03.022 15246235

[B5] Byrd J. A. Anderson R. C. Callaway T. R. Moore R. W. Knape K. D. Kubena L. F. (2003). Effect of experimental chlorate product administration in the drinking water on *Salmonella* Typhimurium contamination of broilers. *Poult. Sci.* 82 1403–1406. 10.1093/ps/82.9.1403 12967253

[B6] Carrasco E. Morales-Rueda A. García-Gimeno R. M. (2012). Cross-contamination and recontamination by *Salmonella* in foods: a review. *Food Res. Int.* 45 545–556. 10.1016/j.foodres.2011.11.004

[B7] CDC (2011). *Salmonella Heidelberg Infections Linked to Ground Turkey.* Available at: https://www.cdc.gov/salmonella/2011/ground-turkey-11-10-2011.html [accessed January 15, 2017].

[B8] CDC (2013). *Morbidity and Mortality Weekly Report: Surveillance for Foodborne Disease Outbreaks — United States, 1998–2008.* Available at: https://www.cdc.gov/mmwr/preview/mmwrhtml/ss6202a1.htm [accessed May 22, 2017]. 23804024

[B9] CDC (2014a). *Multistate Outbreak of Multidrug-Resistant Salmonella Heidelberg Infections Linked to Foster Farms Brand Chicken.* Available at: https://www.cdc.gov/salmonella/heidelberg-10-13/ [accessed January 15, 2017].

[B10] CDC (2014b). *Salmonella Outbreak of Salmonella Heidelberg Infections Linked to Tyson Brand Chicken.* Available at: https://www.cdc.gov/salmonella/heidelberg-01-14/ [accessed January 15, 2017].

[B11] FDA (2016). *CFR - Code of Federal Regulations Title 21: Part 182 – Substances Generally Recognized as Safe: Sec. 182.20. Essential Oils, Oleoresins (Solvent-Free), and Natural Extractives (Including Distillates).* Silver Spring, MD: FDA.

[B12] Foley S. L. Lynne A. M. Nayak R. (2008). *Salmonella* challenges: prevalence in swine and poultry and potential pathogenicity of such isolates. *J. Anim. Sci.* 86(Suppl. 14) E149–E162. 10.2527/jas.2007-0464 17911227

[B13] Folster J. P. Pecic G. Rickert R. Taylor J. Zhao S. Fedorka-Cray P. J. (2012a). Characterization of multidrug-resistant *Salmonella enterica* serovar Heidelberg from a ground turkey-associated outbreak in the United States in 2011. *Antimicrob. Agents Chemother.* 56 3465–3466. 10.1128/AAC.00201-12 22450975 PMC3370747

[B14] Folster J. P. Pecic G. Singh A. Duval B. Rickert R. Ayers S. (2012b). Characterization of extended-spectrum cephalosporin–resistant *Salmonella enterica* serovar Heidelberg isolated from food animals, retail meat, and humans in the United States 2009. *Foodborne Pathog. Dis.* 9 638–645. 10.1089/fpd.2012.1130 22755514 PMC4620655

[B15] FSIS (2002). *Potential Scientific Parameters that must be Considered to Establish Global Dates for Refrigerated Ready-to-Eat Foods.* Available at: https://www.fsis.usda.gov/OPHS/nacmcf/2002/rep_shelflife1.htm [accessed March 11, 2017].

[B16] FSIS (2010). *Compliance Guideline for Controlling Salmonella and Campylobacter in Poultry.* Available at: http://www.fsis.usda.gov/PDF/Compliance_Guide_Controling_Salmonella_Campylobacter_Poultry_0510.pdf

[B17] FSIS (2014). *FSIS Compliance Guide: Modernization of Poultry Slaughter Inspection: Chilling Requirements.* Washington, DC: Food Safety and Inspection Service.

[B18] FSIS (2015). *DRAFT FSIS Compliance Guideline for Controlling Salmonella and Campylobacter in Raw Poultry.* Washington, DC: Food Safety and Inspection Service 1–109.

[B19] Ghosh V. Mukherjee A. Chandrasekaran N. (2013). Ultrasonic emulsification of food-grade nanoemulsion formulation and evaluation of its bactericidal activity. *Ultrason. Sonochem.* 20 338–344. 10.1016/j.ultsonch.2012.08.010 22954686

[B20] GPO (2017). *eCFR — Code of Federal Regulations: Subpart C—Organic Production and Handling Requirements and Subpart D—Labels, Labeling, and Market Information.* Available at: http://www.ecfr.gov/cgi-bin/text-idx?SID=0a4045faa17f9676c258733eb6f1b9e7&mc=true&node=pt7.3.205&rgn=div5#sp7.3.205.c [accessed January 15, 2017].

[B21] Hyldgaard M. Mygind T. Meyer R. L. (2012). Essential oils in food preservation: mode of action, synergies, and interactions with food matrix components. *Front. Microbiol.* 3:12. 10.3389/fmicb.2012.00012 22291693 PMC3265747

[B22] Ingham S. C. Fanslau M. A. Burnham G. M. Ingham B. H. Norback J. P. Schaffner D. W. (2007). Predicting pathogen growth during short-term temperature abuse of raw pork, beef, and poultry products: use of an isothermal-based predictive tool. *J. Food Prot.* 70 1446–1456. 10.4315/0362-028X-70.6.1446 17612076

[B23] Jackson B. R. Griffin P. M. Cole D. Walsh K. A. Chai S. J. (2013). *Salmonella enterica* serotypes and food commodities. *Emerg. Infect. Dis.* 19 1239–1244. 10.3201/eid1908.121511 23876503 PMC3739514

[B24] Kim K. Y. Frank J. F. Craven S. E. (1996). Three-dimensional visualization of *Salmonella* attachment to poultry skin using confocal scanning laser microscopy. *Lett. Appl. Microbiol.* 22 280–282. 10.1111/j.1472-765X.1996.tb01161.x 8934786

[B25] Kollanoor Johny A. Darre M. J. Donoghue A. M. Donoghue D. J. Venkitanarayanan K. (2010). Antibacterial effect of *trans*-cinnamaldehyde, eugenol, carvacrol, and thymol on *Salmonella* Enteritidis and *Campylobacter jejuni* in chicken cecal contents *in vitro*. *J. Appl. Poult. Res.* 19 237–244. 10.3382/japr.2010-00181

[B26] Kollanoor Johny A. Darre M. J. Hoagland T. A. Schreiber D. T. Donoghue A. M. Donoghue D. J. (2008). Antibacterial effect of trans-cinnamaldehyde on *Salmonella* Enteritidis and *Campylobacter jejuni* in chicken drinking water. *J. Appl. Poult. Res.* 17 490–497. 10.3382/japr.2008-00051

[B27] Kollanoor-Johny A. Mattson T. Baskaran S. A. Amalaradjou M. A. Babapoor S. March B. (2012a). Reduction of *Salmonella enterica* serovar Enteritidis colonization in 20-day-old broiler chickens by the plant-derived compounds *trans*-cinnamaldehyde and eugenol. *Appl. Environ. Microbiol.* 78 2981–2987. 10.1128/AEM.07643-11 22327574 PMC3318785

[B28] Kollanoor-Johny A. Upadhyay A. Baskaran S. A. Upadhyaya I. Mooyottu S. Mishra N. (2012b). Effect of therapeutic supplementation of the plant compounds *trans*-cinnamaldehyde and eugenol on *Salmonella enterica* serovar Enteritidis colonization in market-age broiler chickens. *J. Appl. Poult. Res.* 21 816–822. 10.3382/japr.2012-00540

[B29] Lanciotti R. Gianotti A. Patrignani F. Belletti N. Guerzoni M. Gardini F. (2004). Use of natural aroma compounds to improve shelf-life and safety of minimally processed fruits. *Trends Food Sci. Technol.* 15 201–208. 10.1016/j.tifs.2003.10.004

[B30] Landry K. S. Chang Y. McClements D. J. McLandsborough L. (2014). Effectiveness of a novel spontaneous carvacrol nanoemulsion against *Salmonella enterica* Enteritidis and *Escherichia coli* O157: H7 on contaminated mung bean and alfalfa seeds. *Int. J. Food Microbiol.* 187 15–21. 10.1016/j.ijfoodmicro.2014.06.030 25033425

[B31] Lee N. Y. Park S. Y. Kang I. S. Ha S. D. (2014). The evaluation of combined chemical and physical treatments on the reduction of resident microorganisms and *Salmonella* Typhimurium attached to chicken skin. *Poult. Sci.* 93 208–215. 10.3382/ps.2013-03536 24570441

[B32] Lillard H. S. (1990). The impact of commercial processing procedures on the bacterial contamination and cross-contamination of broiler carcasses. *J. Food Prot.* 53 202–207. 10.4315/0362-028X-53.3.202 31018399

[B33] Logue C. M. Sherwood J. S. Elijah L. M. Olah P. A. Dockter M. R. (2003). The incidence of *Campylobacter* spp. on processed turkey from processing plants in the midwestern United States. *J. Appl. Microbiol.* 95 234–241. 10.1046/j.1365-2672.2003.01969.x 12859753

[B34] Nagel G. M. Bauermeister L. J. Bratcher C. L. Singh M. McKee S. R. (2013). *Salmonella* and *Campylobacter* reduction and quality characteristics of poultry carcasses treated with various antimicrobials in a post-chill immersion tank. *Int. J. Food Microbiol.* 165 281–286. 10.1016/j.ijfoodmicro.2013.05.016 23800739

[B35] Nair D. Thomas J. V. Kollanoor-Johny A. (2016). “*Propionibacterium freudenreichii* reduces cecal colonization of multidrug-resistant *Salmonella* Heidelberg in turkey poults,” in *Proceedings of the Poultry Science Association 105th Annual Meeting Abstracts (Schwean-Lardner)* New Orleans 21–22.

[B36] Nair D. V. T. Kiess A. Nannapaneni R. Schilling W. Sharma C. S. (2015). The combined efficacy of carvacrol and modified atmosphere packaging on the survival of *Salmonella, Campylobacter jejuni* and lactic acid bacteria on turkey breast cutlets. *Food Microbiol.* 49 134–141. 10.1016/j.fm.2015.01.010 25846923

[B37] Nair D. V. T. Nannapaneni R. Kiess A. Schilling W. Sharma C. S. (2014). Reduction of *Salmonella* on turkey breast cutlets by plant-derived compounds. *Foodborne Pathog. Dis.* 11 981–987. 10.1089/fpd.2014.1803 25405806

[B38] Nazzaro F. Fratianni F. De Martino L. Coppola R. De Feo V. (2013). Effect of essential oils on pathogenic bacteria. *Pharmaceuticals* 6 1451–1474. 10.3390/ph6121451 24287491 PMC3873673

[B39] Nchez M. X. S. Fluckey W. M. Brashears M. M. Mckee S. R. (2002). Antibiotic susceptibility of *Campylobacter* spp. and *Salmonella* spp. in broilers processed in air-chilled and immersion-chilled environments. *J. Food Prot.* 65 948–956. 10.4315/0362-028X-65.6.948 12092727

[B40] Poppe C. Martin L. C. Gyles C. L. Reid-Smith R. Boerlin P. McEwen S. A. (2005). Acquisition of resistance to extended-spectrum cephalosporins by *Salmonella enterica* subsp. *enterica* serovar Newport and *Escherichia coli* in the turkey poult intestinal tract. *Appl. Environ. Microbiol.* 71 1184–1192. 10.1128/AEM.71.3.1184-1192.2005 15746317 PMC1065184

[B41] Russell S. M. (2008). Chemical residuals in the environment and on chicken carcasses associated with scalding chickens in an acidic, copper sulfate-based commercial sanitizer during poultry processing. *J. Food Prot.* 71 226–230. 10.4315/0362-028X-71.1.226 18236690

[B42] Salehi S. Howe K. Lawrence M. L. Brooks J. P. Bailey R. H. Karsi A. (2017). *Salmonella enterica* Serovar Kentucky flagella are required for broiler skin adhesion and Caco-2 cell invasion. *Appl. Environ. Microbiol.* 83:e02115-16. 10.1128/AEM.02115-16 27793824 PMC5203615

[B43] SAS Institute (2004). *SAS/STAT 9.1 User’s Guide.* Available at: http://books.google.com/books?id=2oEQyOIyCbUC&pgis=1

[B44] Seo K. Holt P. Gast R. (2000). Combined effect of antibiotic and competitive exclusion treatment on *Salmonella* Enteritidis fecal shedding in molted laying hens. *J. Food Prot.* 63 545–548. 10.4315/0362-028X-63.4.545 10772224

[B45] Seo K. H. Frank J. F. (1999). Attachment of *Escherichia coli* O157:H7 to lettuce leaf surface and bacterial viability in response to chlorine treatment as demonstrated by using confocal scanning laser microscopy. *J. Food Prot.* 62 3–9. 10.4315/0362-028X-62.1.3 9921820

[B46] Shah D. H. Paul N. C. Sischo W. C. Crespo R. Guard J. (2017). Population dynamics and antimicrobial resistance of the most prevalent poultry-associated *Salmonella* serotypes. *Poult. Sci.* 96 687–702. 10.3382/ps/pew342 27665007

[B47] Sohaib M. Anjum F. M. Arshad M. S. Rahman U. U. (2015). Postharvest intervention technologies for safety enhancement of meat and meat based products; a critical review. *J. Food Sci. Technol.* 53 19–30. 10.1007/s13197-015-1985-y 26787929 PMC4711421

[B48] Surendran Nair M. Lau P. Belskie K. Fancher S. Chen C.-H. Karumathil D. P. (2016). Potentiating the heat inactivation of *Escherichia coli* O157: H7 in ground beef patties by natural antimicrobials. *Front. Microbiol.* 7:15. 10.3389/fmicb.2016.00015 26870000 PMC4735374

[B49] Surendran-Nair M. Kollanoor-Johny A. Ananda-Baskaran S. Norris C. Lee J.-Y. Venkitanarayanan K. (2016). Selenium reduces enterohemorrhagic *Escherichia coli* O157: H7 verotoxin production and globotriaosylceramide receptor expression on host cells. *Future Microbiol.* 11 745–756. 10.2217/fmb.16.16 27191971

[B50] Surendran Nair M. Upadhyaya I. Amalaradjou M. A. R. Venkitanarayanan K. (2017). “Antimicrobial food additives and disinfectants,” in *Foodborne Pathogens and Antibiotic Resistance* ed. Singh O. V. (Hoboken, NJ: John Wiley & Sons, Inc.) 275–301. 10.1002/9781119139188.ch12

[B51] Suzuki É. Y. Baptista E. B. Resende Do Carmo A. M. Miranda Chaves M. D. G. A. Chicourel E. L. Barbosa Raposo N. R. (2014). Potential of the essential oil from *Pimenta pseudocaryophyllus* as an antimicrobial agent. *Acta Pharm.* 64 379–385. 10.2478/acph-2014-0024 25296683

[B52] Tamblyn K. C. Conner D. E. (1997). Bactericidal activity of organic acids against *Salmonella* Typhimurium attached to broiler chicken skin. *J. Food Prot.* 60 629–633. 10.4315/0362-028X-60.6.629 31195563

[B53] Tamblyn K. C. Conner D. E. Bilgili S. F. (1997). Utilization of the skin attachment model to determine the antibacterial efficacy of potential carcass treatments. *Poult. Sci.* 76 1318–1323. 10.1093/ps/76.9.1318 9276898

[B54] Tan S. M. Lee S. M. Dykes G. A. (2014). Fat contributes to the buffering capacity of chicken skin and meat but enhances the vulnerability of attached *Salmonella* cells to acetic acid treatment. *Food Res. Int.* 66 417–423. 10.1016/j.foodres.2014.10.007

[B55] Upadhyaya I. Upadhyay A. Kollanoor-Johny A. Darre M. Venkitanarayanan K. (2013). Effect of plant derived antimicrobials on *Salmonella* Enteritidis adhesion to and invasion of primary chicken oviduct epithelial cells *in vitro* and virulence gene expression. *Int. J. Mol. Sci.* 14 10608–10625. 10.3390/ijms140510608 23698782 PMC3676857

[B56] Venkitanarayanan K. Kollanoor-Johny A. Darre M. J. Donoghue A. M. Donoghue D. J. (2013). Use of plant-derived antimicrobials for improving the safety of poultry products. *Poult. Sci.* 92 493–501. 10.3382/ps.2012-02764 23300319

[B57] Waldroup A. L. (1996). Contamination of raw poultry with pathogens. *Worlds Poult. Sci. J.* 52 20–25. 10.1079/Wps19960002

[B58] WHO (2009). *Microbiological Risk Assessment Series-19: Salmonella and Campylobacter in Chicken Meat.* Available at: http://www.who.int/foodsafety/publications/micro/MRA19.pdf [accessed August 21, 2017].

[B59] Yang H. Li Y. B. Johnson M. G. (2001). Survival and death of *Salmonella* Typhimurium and *Campylobacter jejuni* in processing water and on chicken skin during poultry scalding and chilling. *J. Food Prot.* 64 770–776. 10.4315/0362-028X-64.6.770 11403124

[B60] Young S. D. Olusanya O. Jones K. H. Liu T. Liljebjelke K. A. Hofacre C. L. (2007). *Salmonella* incidence in broilers from breeders vaccinated with live and killed *Salmonella*. *J. Appl. Poult. Res.* 16 521–528. 10.3382/japr.2007-00009

